# Validation of the mTICCS Score as a Useful Tool for the Early Prediction of a Massive Transfusion in Patients with a Traumatic Hemorrhage

**DOI:** 10.3390/jcm9040945

**Published:** 2020-03-30

**Authors:** Klemens Horst, Rachel Lentzen, Martin Tonglet, Ümit Mert, Philipp Lichte, Christian D. Weber, Philipp Kobbe, Nicole Heussen, Frank Hildebrand

**Affiliations:** 1 Department of Trauma and Reconstructive Surgery, University Hospital, RWTH 52074 Aachen, Germany; rlentzen@ukaachen.de (R.L.); umert@ukaachen.de (Ü.M.); plichte@ukaachen.de (P.L.); chrweber@ukaachen.de (C.D.W.); pkobbe@ukaachen.de (P.K.); fhildebrand@ukaachen.de (F.H.); 2 Department of Emergency, Liege University Hospital, Domaine du Sart Tilman, 4000 Liege, Belgium; tongletm@yahoo.com; 3 Department of Medical Statistics, RWTH Aachen University, 52074 Aachen, Germany; nheussen@ukaachen.de; 4 Medical School, Sigmund Freud Private University, 1020 Vienna, Austria

**Keywords:** mTICCS, TICCS, massive transfusion, shock, multiple trauma, polytrauma, bleeding, transfusion

## Abstract

The modified Trauma-Induced Coagulopathy Clinical Score (mTICCS) presents a new scoring system for the early detection of the need for a massive transfusion (MT). While validated in a large trauma cohort, the comparison of mTICCS to established scoring systems is missing. This study therefore validated the ability of six scoring systems to stratify patients at risk for an MT at an early stage after trauma. A dataset of severely injured patients (ISS ≥ 16) derived from the database of a level I trauma center (2010–2015) was used. Scoring systems assessed were Trauma-Associated Severe Hemorrhage (TASH) score, Prince of Wales Hospital (PWH) score, Larson score, Assessment of Blood Consumption (ABC) score, Emergency Transfusion Score (ETS), and mTICCS. Demographics, diagnostic data, mechanism of injury, injury pattern (graded by AIS), and outcome (length of stay, mortality) were analyzed. Scores were calculated, and the area under the receiver operating characteristic curves (AUCs) were evaluated. From the AUCs, the cut-off point with the best relationship of sensitivity-to-specificity was used to recalculate sensitivity, specificity, positive predictive values (PPV), and negative predictive values (NPV). A total of 479 patients were included; of those, blunt trauma occurred in 92.3% of patients. The mean age of patients was 49 ± 22 years with a mean ISS of 25 ± 29. The overall MT rate was 8.4% (*n* = 40). The TASH score had the highest overall accuracy as reflected by an AUC of 0.782 followed by the mTICCS (0.776). The ETS was the most sensitive (80%), whereas the TASH score had the highest specificity (82%) and the PWH score had the lowest (51.83%). At a cut-off > 5 points, the mTICCS score showed a sensitivity of 77.5% and a specificity of 74.03%. Compared to sophisticated systems, using a higher number of weighted variables, the newly developed mTICCS presents a useful tool to predict the need for an MT in a prehospital situation. This might accelerate the diagnosis of an MT in emergency situations. However, prospective validations are needed to improve the development process and use of scoring systems in the future.

## 1. Introduction

Traumatic hemorrhage still represents a challenging problem in injured patients [1,2]. In this context, uncontrolled bleeding is found in about 50% of patients, deceasing in the first 48 hours after trauma, thereby presenting one of the leading causes of early posttraumatic death. The main sources of severe bleeding are thoracic, abdominal, and pelvic injuries [3,4]. Although early laboratory diagnostics, including standard coagulation parameters, ROTEM®, and implementation of massive transfusion (MT) protocols, have been found to improve the outcome, prediction, and early identification of patients which need MT is still difficult [5,6,7]. For this purpose, several scores using clinical parameters, laboratory results, and imaging have been introduced and validated [8,9]. However, some of the relevant scoring parameters are not available before diagnostics in the emergency room have been finalized, which negatively affects their clinical utility for prediction of a necessary MT. In 2014, Tonglet et al. introduced the “Trauma-Induced Coagulopathy Clinical Score” (TICCS), which is based on the general injury severity assessment, blood pressure, and extent of bodily injury (Table 1) [10]. Due to its simplicity, this calculation is feasible on scene and allows for the timely identification of patients in need of the preclinical start of damage control resuscitation (DCR) and later, possibly, an MT. Modified Trauma-Induced Coagulopathy Clinical Score (mTICCS) was validated in 2017 in a population of 33,385 trauma patients [11]. The present work aims to compare the newly developed mTICCS with established and frequently used scoring systems to identify patients at risk for needing an MT.

## 2. Experimental Section

Severely injured patients (ISS ≥ 16) that were consecutively admitted to our trauma center (Level 1) between 2010 and 2015 were included in this study. Demographic data, as well as laboratory parameters (hemoglobin (Hb), lactate (Lac), partial thromboplastin time (PTT), International Normalized Ratio (INR)), and imaging data (focused assessment with sonography for trauma (FAST), X-ray, and CT), were collected. Furthermore, the mechanism of injury, injury pattern (graded by AIS), and outcome (length of stay, mortality) were recorded. Due to the retrospective nature of this study, ethical approval was not required.

### 2.1. Scores

Only scores including parameters that could be reproduced by the data captured in the hospital information system were considered. An MT was defined as the administration of 10 or more units of packed red blood cells (pRBC) in the first 24 h after arrival at the emergency room (ER). Scores were raised by two investigators (KH; RL). In case of discrepancies, results were discussed until consensus was achieved. 

#### 2.1.1. Trauma-Associated Severe Hemorrhage (TASH) Score

To identify the risk for needing an MT, the TASH score uses seven independent, weighted variables: systolic blood pressure, sex, hemoglobin, focused assessment for the sonography of trauma (FAST), heart rate, base excess (BE), and extremity or pelvic fractures. Using a logistic function, the TASH score is transformed into the probability of an MT. Consequently, a score ≥ 16 points indicates a > 50% probability of an MT. Furthermore, a score ≥ 27 points is associated with a 100% predicted and obtained risk for an MT [12,13].

#### 2.1.2. Assessment of Blood Consumption (ABC) Score

The ABC score uses parameters: penetrating trauma mechanism, systolic blood pressure ≤ 90 mmHg on ER arrival, heart rate ≥ 120 bpm on ER arrival, and positive FAST examination, which are available during the first few minutes after hospital admission of a trauma patient, to assess the risk for an MT [14].

#### 2.1.3. Larson Score

The Larson score is based on the “Joint Theater Trauma Registry” transfusion database, which includes data of US service personnel injured in combat scenarios during overseas deployment. The Larson score includes heart rate, systolic blood pressure, hemoglobin, and base deficit to identify the probability of an MT [15].

#### 2.1.4. Prince of Wales Hospital (PWH) Score 

The PWH score is based on data from 1891 civilian trauma patients admitted to The Prince of Wales Hospital in Hong Kong. Statistical analyses identified seven parameters: heart rate ≥ 120 bpm, systolic blood pressure ≤ 90 mmHg, Glasgow coma scale ≤ 8, displaced pelvic fracture, computer tomography (CT) scan or FAST-positive for fluid, base deficit > 5 mmol/L, hemoglobin ≤ 7 g/dL, and hemoglobin 7.1–10.0 g/dL, to predict the need for an MT [16]. 

#### 2.1.5. Emergency Transfusion (ET) Score

Based on data from 1103 patients, nine parameters, with different predictive values indicating the need for a blood transfusion, were chosen for the ET score, including systolic blood pressure (SBP) < 90 mmHg or SBP 90–120 mmHg, free fluid on the abdominal ultrasound, clinically unstable pelvic ring fracture, aged 20–60 years or > 60 years, admission from scene of accident, traffic accident, and fall from > 3 m. Score values range from 1 (0.7% probability of an MT) to 9.5 (97% probability of an MT) points [17].

#### 2.1.6. Modified Trauma-Induced Coagulopathy Clinical Score (mTICCS)

The mTICCS is a modified version of the TICCS [10]. There are three assessment criteria that form the score: the assessment of general severity, blood pressure, and the extent of body injury (Table 1). The original TICCS separated critical patients being admitted to the emergency room and noncritical patients without activation of the emergency room. If the emergency room was activated, 2 points were assigned, otherwise the patient was graded with 0 points. As severely injured patients are generally treated in emergency rooms, all patients were automatically assigned 2 points. Additionally, mTICCS does not discriminate between left and right extremity injuries but rather whether the injury affected the upper (left and/or right) or lower (left and/or right) extremities. In the modified scoring system, 1 point was attributed to a severe injury of the upper extremities and 1 point for a severe injury of the lower extremities. 

### 2.2. Statistical Analyses

Demographic data as well as injury mechanisms and pattern were described in tables by median and range or frequency and percentage. Comparisons between MT and no MT groups were performed by Wilcoxon rank-sum-test for continuous data or Chi-square-test. Results are presented as *p*-values. 

mTICCS was compared to TASH, ABC, Larson, PWH, and ETS scores to evaluate its diagnostic accuracy. The different scores were evaluated with the need for an MT within the first 24 h after admission. The area under the receiver operating characteristic curve (AUC) was calculated for each score. The Youden Index was used to determine the cut-off point at which sensitivity and specificity were of equal diagnostic importance. The Youden Index specifies the optimal cut-off point by maximizing the difference between true positive and false positive ratings over all possible cut-off points. Prevalence, sensitivity, specificity, positive predicted values (PPV), and negative predicted values (NPV) with corresponding 95% confidence limits (95% CI) were used for the comparison of scores.

The AUC of the mTICCS was compared to the AUCs of TASH, ABC, Larson, PWH, and ETS scores to assess and compare the overall diagnostic accuracy. Results were reported as the AUC value with the corresponding 95% confidence limits and *p*-values. For all comparisons, the significance level was set to 5%, and due to the explorative nature of this study no adjustment was made to the significance level for multiple testing. All analyses were performed by SPSS 25.0 (IBM®, New York, NY, USA) and MedCalc® version 18.9.1 (MedCalc software, Ostend, Belgium).

## 3. Results

### 3.1. General Data 

In total, 479 patients were included in this study. Demographic data, injury mechanisms, as well as mortality rates are displayed in Table 2. Overall, patients were discharged after 21 (29) days, of which they spent 14 (31) days in the intensive care unit. On average, patients were ventilated for 194 (398) hours. The overall MT rate was 8.4% (*n* = 40).

### 3.2. AUC Analysis 

**Figure 1:**
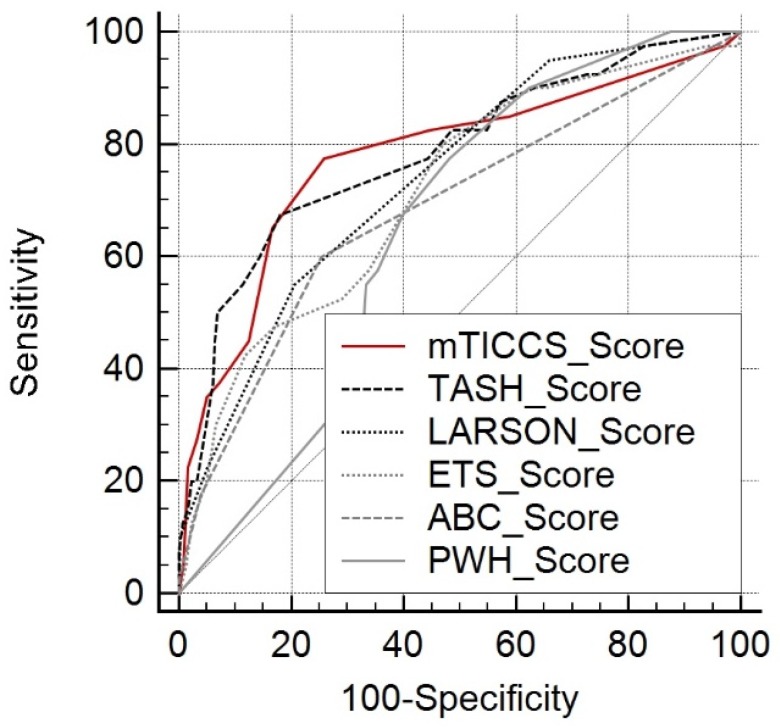
Area under the receiver operating characteristic curves (AUCs) for the mTICCS, Trauma-Associated Severe Hemorrhage (TASH), Assessment of Blood Consumption (ABC), Larson, Prince of Wales Hospital (PWH), and Emergency Transfusion Score (ETS) scores.

For the tested scores, AUC analysis revealed values between 0.648 (PWH) and 0.782 (TASH) (Table 3; Figure 1). mTICCS had a significantly larger AUC than PWH (0.776, 95% CI: (0.736; 0.812) vs. 0.648, 95% CI: (0.603; 0.691); *p* = 0.0103). However, no significant difference was identified between the AUC of mTICCS and the AUCs of the other scores (Table 3). 

### 3.3. Prevalence, Sensitivity, Specificity, Positive, and Negative Predictive Values 

Sensitivity was highest for ETS, followed by mTICCS, PWH, TASH, ABC, and Larson, respectively. However, specificity was highest for TASH, followed by Larson, ABC, mTICCS, ETC, and PWH (Table 4). TASH also presented with the highest PPV, while NPV was highest for mTICCS (Table 4).

Considering mTICCS as screening tool, 145 were identified at risk of MT (145 test positives among 479 screend patients), and among these 145 patients, 31 went on to need MT (PPV of 21%). In addition, of the 40 patients needing MT in our sample, 31 were correctly identified by mTICCS (sensitivity of 77.50%) while 9 patients were missed (false negative). On the other hand, of the 439 patients who do not need MT, 325 were correctly identified (specificity of 74.03%) while 114 were missed (false positive).

## 4. Discussion

Although early management of acute coagulopathy in trauma victims improves the outcome, timely identification and prediction of a trauma-induced hemorrhage and the associated need for an MT remains challenging [18,19]. Therefore, the present study compared the newly developed and easily applicable mTICCS to five established scoring algorithms in order to classify the relevance of mTICCS as a prediction method for an MT. Our results show that the AUC of the mTICCS was comparable to the TASH, Larson, ABC, and ETS scores and superior to the PHW score. mTICCS is calculated with the lowest number of assessment criteria, which are also easily accessible and gained early on in evaluation. These findings make mTICCS highly interesting for a clinical routine, as the assessment criteria of general trauma severity, blood pressure, and extent of body injury do not need sophisticated diagnostic tools and can easily be scored by paramedics as well as hospital staff. 

In general, one could assume that more complex scores are superior for prediction of an MT when compared to more compact systems. Accordingly, the TASH score [12], which includes demographic data, physical variables, laboratory results, injury patterns, and sonography, was shown to represent the gold standard in the prediction of an MT with the highest AUC (0.889) when compared to the PWH (0.860) and the score of Vandromme et al (0.840) in a trauma cohort of 5147 patients [9]. The authors of this study concluded that weighted and more sophisticated systems like TASH and PWH scores, which include more and often similar variables, performed better than those that use simple, nonweighted models [9]. However, the PWH score did not perform well in our study and presented with a significantly lower AUC than mTICCS. This is interesting because the PWH score uses comparable components to the TASH score and applies weights to selected individual parameters to stratify patient’ risk for an MT. Similar to the results found in our study, Mitra et al. showed, in a trauma cohort of 1234 Australian patients, that the PWH score was significantly less predictive for an MT than other scores [20]. The PWH score was initially developed and validated in the New Territories of Hong Kong, where 95% of the population are Chinese; therefore, ethical aspects might explain the observed differences between the scoring systems. Similarly, Singhal et al. showed that thromboelastography analysis of Asian and Caucasian patients revealed different hemostatic mechanisms [21]. In this context, Asians presented with significantly lower postoperative factor levels than Caucasians, and baseline comparisons of factor and serum levels revealed that Asians presented with the lowest thrombogenic profiles when compared to other ethnic groups. Further aspects in regard to different hemostatic mechanisms between the two races were confirmed [21,22]. In contrast to the PHW score, the well-performing TASH score was developed and validated in a Caucasian population; therefore, it may be assumed that ethnicity has a significant influence on the relevance of a score to evaluate the probability of an MT. 

In addition to ethnicity, other aspects of the study population’s composition need to be considered when comparing the predictive value of different scores. In this context, Brockamp et al. included patients with an ISS > 9 who survived until ICU admission [9]. Thus, it might be assumed that those scores were tested against a relatively “stable” cohort, with a lower risk for bleeding complications, compared to the scores included in the present study (ISS > 16, admission to shock room). This is supported by a higher percentage of MTs in the present study (8.4%) compared to 5.6%, which was reported by Brockamp et al. [9]. When considering the highly complex TASH and PWH scores, combat zones, as well as civilian scenarios with severely injured patients, recommend more simple tools to stratify patient’ risk for an MT [14,15,17]. Other scores (Larson, ABC, and ET scores) have aimed to address this issue; however, these scores still use either laboratory (e.g., base deficit, hemoglobin) or other diagnostic (e.g., x-ray, FAST) variables, which are probably not available on scene or hamper timely identification of patients in need of the preclinical start of DCR and a future MT. Furthermore, although the data needed to calculate these scores may be available in a relatively short time after hospital admission, it still requires time to collect the data and calculate the score. This calls into question the utility of these scores as “early” identification tools and explains why these scores are not routinely used in clinical practice. 

The original version of TICCS focused on easy and early application and could be determined before hospital admission [10,23]. Due to the known benefits of additional variables, like laboratory parameters or diagnostic imaging procedures, adding to the validity of prediction scores, Swerts et al. hypothesized that using BE and FAST would improve the predictive value of TICCS. Both parameters were chosen because they could theoretically be used in a prehospital setting. However, adding these variables to the original TICCS did not significantly improve the scoring system’s ability to predict the need for an MT [24]. Unfortunately, the authors do not discuss possible reasons for their findings. BE has recently been discussed critically as a possible scoring variable [25], as it reflects the acid-base status and may increase during metabolic and respiratory acidosis and thus influence therapeutic resuscitation, such as fluid loading (chloride-induced acidosis) [26]. Moreover, in regard to additional imaging procedures, Becker et al. observed that the false-negative rate of FAST performed in blunt abdominal trauma patients with a high injury severity score (ISS > 25) is higher than that performed in patients with an ISS < 25 [27]. The authors demonstrated that the sensitivity of FAST is lower among patients with an ISS > 25 (65.1%) than patients with an ISS < 25 (86.4%). Becker at al. concluded that patients with a high ISS are at an increased risk of having ultrasound-occult injuries and have a lower accuracy during their ultrasound examination than patients with a low to moderate ISS [27]. Thus, including FAST into a scoring system for an MT prediction should be discussed carefully. 

The original TICCS was adjusted to the modified version (mTICCS) in regard to the special characteristics of severely injured patients and the need to simplify predictions scores [11]. With this study we were able to show that despite its simplicity, the prognostic value (as indicated by the AUC) of mTICCS was not significantly different than that of the other scores. Thus, mTICCS might provide a useful tool to predict the need for an MT at a very early stage after trauma.

For the present analysis, a single dataset from a Level 1 trauma center was used, including a significant number of severely injured patients. All containing variables for the scoring systems and algorithms could be assessed. Furthermore, detailed data on transfusion practices and the use of blood products were available. However, data presented in the current study were collected from a retrospective database with information collected after hospital admission. Thus, information about prehospital applications of hemostatic agents with a potential influence on the amount of administered pRBCs was not available and might have caused some bias to the results. Furthermore, the score should be used by paramedics, nursing staff in the emergency room, as well as emergency physicians. Due to the retrospective nature of the study, an interrater liability might occur. Taking these shortcomings into account, prospective validation of the presented scores is urgently needed.

## 5. Conclusions

Based on its three assessment criteria, including the assessment of general severity, blood pressure, and extent of bodily injury, the mTICCS showed no significant difference in AUC when compared to the AUCs of other established and more sophisticated scores. Yet, due to mTICCS simple applicability, diagnosis can be made very early after hospital admission or even in the prehospital setting. Thus, mTICCS might provide a new useful diagnostic tool to detect patients with ongoing bleeding in need of an MT. Consequently, the time until therapy is initiated can be reduced. Prospective, follow-up studies are needed to determine the effect of this efficiency on outcome parameters. 

## Figures and Tables

**Table 1:** modified Trauma Induced Coagulopathy Clinical Score (Mticcs) criteria.

Criteria	Points
**General Severity** Admitted into resuscitation room with trauma team activation	2
**Blood Pressure** SBP * fell below 90 mmHg at least once SBP * always above 90 mmHg	5 0
**Extent of Significant Injuries** Head and neck Upper extremity (left or right) Lower extremity (left or right) Torso Abdomen Pelvis	1 1 1 2 2 2
**Total Possible Score**	2–16

* SBP: systolic blood pressure.

**Table 2:** Demographic data, injury mechanisms, and trauma severity.

Demographic Data	No MT *n* = 439	MT *n* = 40	*p*-Value
Age in years median (range)	50 (2–93)	42 (11–82)	0.536 ^†^
Male sex in % (*n*)	70.6 (310)	80.0 (32)	0.209 ^§^
^‡^ ISS median (range)	24 (16–75)	29 (17–59)	0.000 ^†^
^#^ NISS median (range)	27 (16–75)	31 (17–59)	0.011 ^†^
RR * ≤ 90mmHg initial in % (*n*)	4.1 (18)	17.5 (7)	0.001 ^§^
^¥^ BP in total ≤ 24 h median (range)	0 (0–9)	21.5 (10–106)	0.001 ^†^
Overall mortality in % (*n*)	23.5 (103)	52.5 (21)	0.000^§^
**Injury Mechanism (% n)**			
Blunt	92.5 (406)	87.5 (35)	0.072 ^§^
Car	17.3 (76)	15 (6)	0.710 ^§^
Motorbike	11.2 (49)	17.5 (7)	0.232 ^§^
Bicycle	7.5 (33)	0 (0)	0.072 ^§^
Fall < 3 m	22.3 (98)	7.5 (3)	0.028 ^§^
Fall > 3 m	16.2 (71)	20 (8)	0.532 ^§^
Pedestrian	8.2 (36)	20 (8)	0.013 ^§^
Burn	3 (13)	0 (0)	0.270 ^§^
Other	14.1 (62)	20 (8)	0.314 ^§^
**^$^ AIS median (range)**			
Head	3 (0–5)	2 (0–5)	0.193 ^†^
Face	0 (0–3)	0 (0–3)	0.444 ^†^
Thorax	2 (0–5)	3 (0–5)	0.132 ^†^
Abdomen	0 (0–5)	2 (0–5)	0.000 ^†^
Extremities	2 (0–5)	3 (0–4)	0.000 ^†^
External	0 (0–6)	0 (0–2)	0.624 ^†^

* RR = blood pressure by Riva-Rocci in mmHg; ^¥^ BP = blood products; ^‡^ ISS = Injury Severity Scale; ^#^ NISS = New Injury Severity Scale; ^$^ AIS = Abbreviated Injury Score. ^†^ Wilcoxon rank-sum-test for continuous data, ^§^ Chi-square-test.

**Table 3:** Comparative area under the curve (AUC) analysis.

Score	mTICCS	TASH	ABC	Larson	PWH	ETS
AUC	0.776	0.782	0.684	0.740	0.648	0.713
95% CI *	0.736; 0.812	0.743; 0.819	0.641; 0.726	0.698; 0.779	0.603; 0.691	0.670; 0.753
Cut-off	> 5	> 8	> 0	> 1	>2	> 2.5
*p*-value for the comparison of AUC to AUC of mTICCS		0.8852	0.0756	0.3839	0.0103	0.0804

* CI (confidence interval).

**Table 4:** Prevalence, sensitivity, specificity, positive predictive value (PPV), and negative predictive value (NPV).

Score	mTICCS	TASH	ABC	Larson	PWH	ETS
**Prevalence (%)**	8.35	8.35	8.35	8.35	8.35	8.35
**Sensitivity (%)**	77.50	67.50	60.00	55.00	77.50	80.00
95% CI *	61.5; 89.2	50.9; 81.4	43.3; 75.1	38.5; 70.7	61.5; 89.2	64.4; 90.9
**Specificity (%)**	74.03	82.00	74.56	79.50	51.83	52.97
95% CI *	69.7; 78.1	78.1; 85.5	70.3; 78.6	75.4; 83.2	47.0; 56.5	48.2; 57.7
**PPV (%)**	21.4	25.5	17.8	19.6	12.8	13.4
95% CI *	17.8; 25.5	20.3; 31.4	13.8; 22.6	14.9; 25.5	10.8; 15.1	11.4; 15.7
**NPV (%)**	97.3	96.5	95.3	95.1	96.2	96.7
95% CI *	95.3; 98.5	94.5; 97.7	93.3; 96.8	93.2; 96.5	93.4; 97.8	93.9; 98.2

* CI (confidence interval).
